# Impact of cardiometabolic risk factors on hepatocellular carcinoma incidence in patients with chronic hepatitis B: A retrospective cohort study

**DOI:** 10.1371/journal.pone.0341366

**Published:** 2026-01-23

**Authors:** Chanavee Toh, Kedsiree Sanit, Pimsiri Sripongpun, Naichaya Chamroonkul, Suthat Liangpunsakul, Teerha Piratvisuth, Apichat Kaewdech

**Affiliations:** 1 Division of Digital Innovation and Data Analytics, Faculty of Medicine, Prince of Songkla University, Songkhla, Thailand; 2 Gastroenterology and Hepatology Unit, Division of Internal Medicine, Faculty of Medicine, Prince of Songkla University, Songkhla, Thailand; 3 Division of Gastroenterology and Hepatology, Department of Medicine, Indiana University School of Medicine, Indianapolis, Indiana, United States of America; 4 Department of Biochemistry and Molecular Biology, Indiana University School of Medicine, Indianapolis, Indiana, United States of America; 5 Roudebush Veterans’ Administration Medical Center, Indianapolis, Indiana, United States of America; 6 NKC Institute of Gastroenterology and Hepatology, Faculty of Medicine, Prince of Songkla University, Songkhla, Thailand; Mayo Clinic Florida: Mayo Clinic's Campus in Florida, UNITED STATES OF AMERICA

## Abstract

**Background and aims:**

Chronic hepatitis B virus (HBV) infection remains a major global health burden and a leading cause of hepatocellular carcinoma (HCC). While cirrhosis is a well-established risk factor, the contributions of metabolic dysfunction-associated steatotic liver disease (MASLD) and cardiometabolic risk factors (CMRFs) are less clearly defined. This study aimed to evaluate the impact of MASLD and CMRFs on HCC risk in patients with chronic hepatitis B (CHB).

**Methods:**

We conducted a retrospective cohort study of CHB patients at Songklanagarind Hospital between 2011 and 2021, excluding those diagnosed with HCC within six months of follow-up. Clinical and imaging data were analyzed. Cumulative HCC incidence was estimated using Nelson-Aalen plots. Multivariable Cox regression was used to identify independent predictors. The aMAP score was evaluated in subgroups with obesity, CMRFs, and MASLD.

**Results:**

Among 4,944 patients, 151 (3.1%) developed HCC. Cirrhosis (adjusted hazard ratio [aHR] 7.22), obesity (aHR 1.85), and male sex (aHR 1.78) were independent risk factors. Statin use (aHR 0.43), higher platelet count (aHR 0.62), and higher albumin (aHR 0.64) were protective. Diabetes and hypertension showed nonsignificant trends, and steatosis and dyslipidemia without statins were not significantly associated with HCC. Risk increased with the number of CMRFs. The aMAP score showed good discrimination in patients with obesity (C-index 0.82), CMRFs (0.79), MASLD (0.74), and in the non-cirrhotic MASLD and non-MASLD (0.69 and 0.71, respectively).

**Conclusions:**

Cirrhosis, male sex, and obesity were key HCC risk factors. The aMAP score effectively stratified HCC risk among metabolically at-risk CHB patients.

## Introduction

Chronic hepatitis B virus (HBV) infection remains a significant global health burden, affecting an estimated 254 million people worldwide in 2022 [1]. It is a leading cause of hepatocellular carcinoma (HCC), particularly in high-prevalence regions such as East and Southeast Asia [2–4]. Although the widespread use of nucleos(t)ide analogs has effectively suppressed HBV replication and reduced liver-related complications [5], HCC continues to develop in virologically controlled patients, suggesting the involvement of non-viral host factors [6].

In parallel with the evolving landscape of liver disease, cardiometabolic risk factors (CMRFs), including obesity, type 2 diabetes mellitus (T2DM), hypertension, and dyslipidemia, have emerged as independent contributors to liver fibrosis and HCC, particularly among patients with metabolic dysfunction-associated steatotic liver disease (MASLD) [7–11]. MASLD, formerly known as non-alcoholic fatty liver disease (NAFLD), reflects a broader metabolic phenotype and allows for coexistence with chronic viral hepatitis, including HBV. As a result, an increasing number of patients with chronic hepatitis B (CHB) now present with coexisting MASLD or CMRFs, especially in Asia, where the prevalence of metabolic disorders is rising despite lower average body mass index (BMI) thresholds [12].

The relationship between MASLD or CMRFs and HCC risk in patients with CHB is complex and remains controversial [13,14]. Some studies suggest a synergistic effect, indicating that hepatic steatosis or diabetes may increase HCC risk even in non-cirrhotic CHB [15,16]. For example, a large Taiwanese cohort reported a significantly higher 10-year HCC incidence in CHB patients with three or more metabolic risk factors (13.6% vs. 4.8%) [7]. Moreover, existing HCC risk prediction models such as PAGE-B and aMAP were developed without accounting for MASLD or CMRFs status [17,18]. This raises concerns that current surveillance guidelines may underestimate HCC risk in metabolically vulnerable CHB patients, particularly as population aging and metabolic syndrome become more prominent in HBV-endemic countries.

In summary, although cirrhosis remains the dominant predictor of HCC, growing evidence suggests that MASLD and CMRFs may play independent or additive roles in hepatocarcinogenesis among patients with CHB [7,15,19]. These metabolic factors may contribute to liver carcinogenesis through mechanisms such as insulin resistance, chronic inflammation, lipotoxicity, and hepatic stellate cell activation [3,5]. However, data from real-world Asian populations remain limited, and many existing studies have either focused on Western cohorts or lack detailed metabolic profiling.

This study aims to address this critical gap by investigating the impact of MASLD and CMRFs on HCC development among patients with CHB. The findings may inform refinements to HCC surveillance strategies and enhance the performance of existing predictive models, such as aMAP and PAGE-B, by incorporating metabolic risk factors, thereby more accurately reflecting the evolving epidemiology of HCC in the HBV era.

## Materials and methods

### Study design and population

This retrospective cohort study was conducted at Songklanagarind Hospital, a tertiary care center in Hatyai, Southern Thailand. The inclusion period for CHB patients was January 1, 2011 to December 31, 2021. All enrolled patients were subsequently followed for HCC development through December 31, 2024, which served as the censor date for outcome assessment. The study included adult patients (aged ≥18 years) with confirmed chronic HBV infection, defined by persistent hepatitis B surface antigen (HBsAg) positivity for at least six months. We included patients with and without CMRFs, such as diabetes mellitus, hypertension, dyslipidemia, and overweight/obesity, in order to compare the outcomes of those with and without metabolic dysfunction and evaluate the effect of CMRFs on the risk of HCC.

Patients were excluded if they had a diagnosis of HCC prior to or within six months of HBV diagnosis, co-infection with other hepatitis viruses (e.g., hepatitis C or D), HIV infection, or evidence of other chronic liver diseases such as alcohol-associated liver disease, Wilson’s disease, autoimmune hepatitis, or cirrhosis due to non-HBV etiologies.

The study protocol was approved by the Institutional Review Board and Ethics Committee of the Faculty of Medicine, Prince of Songkla University, Hat Yai, Thailand (REC: 68-211-14-1). This study was conducted in accordance with the ethical principles outlined in the Declaration of Helsinki (1975). Informed consent was waived owing to the retrospective nature of the study. All data used in this study were accessed on [10, June, 2025] through the Health Information System (HIS) of Songklanagarind Hospital. The authors had access only to de-identified data, and no information that could directly identify individual participants was available during or after data collection. Patient confidentiality was strictly maintained in accordance with institutional and international ethical guidelines.

### Assessment of cardiometabolic risk factors

CMRFs were assessed through a comprehensive review of electronic medical records obtained from the Health Information System (HIS) of Songklanagarind Hospital. Hypertension (HT) was defined by a systolic blood pressure (SBP) ≥140 mmHg, a diastolic blood pressure (DBP) ≥90 mmHg on at least one documented occasion, or the use of antihypertensive medications [20]. Diabetes mellitus (DM) was identified by a fasting plasma glucose level ≥126 mg/dL, a hemoglobin A1c (HbA1c) level ≥6.5%, or the use of antidiabetic medications [21]. Prediabetes (PreDM) was classified when fasting glucose was ≥ 100 mg/dL or HbA1c was ≥ 5.7% on at least one occasion [21]. Dyslipidemia (DLP) was defined by one or more of the following criteria: high-density lipoprotein (HDL) ≤40 mg/dL in males or ≤50 mg/dL in females, low-density lipoprotein (LDL) ≥160 mg/dL, triglycerides ≥150 mg/dL, total cholesterol ≥200 mg/dL, or the use of statins or other lipid-lowering agents [22]. Body mass index (BMI) thresholds were used to define overweight (BMI ≥ 23 kg/m²) and obesity (BMI ≥ 25 kg/m²), consistent with Asian-specific criteria [23].

Hepatic steatosis was identified based on imaging evidence of fat accumulation in the liver, as detected by ultrasonography, computed tomography (CT), or magnetic resonance imaging (MRI). In addition, steatosis was defined by a controlled attenuation parameter (CAP) value ≥248 dB/m, measured using transient elastography (FibroScan) [24]. To ensure temporal relevance, the diagnosis of steatosis had to be established within 90 days of the initial HBV diagnosis. For patients with multiple assessments, a prespecified modality hierarchy reflecting diagnostic sensitivity was applied (MRI ≈ CAP > CT ≈ ultrasound) [25]. In case of discordant results from different modalities, the diagnosis from the higher-sensitivity modality was used. If results originated from modalities of equal sensitivity, the value closest to the index date was chosen. The primary analysis followed a complete-case approach; robustness checks were conducted where appropriate. MASLD was diagnosed in patients with hepatic steatosis and at least one of the five CMRFs outlined in the MASLD consensus guidelines in the absence of significant alcohol consumption, defined as <30 g/day for men and <20 g/day for women [26–29].

### Data collection

Data were retrospectively collected from the hospital’s HIS. Baseline clinical characteristics included demographic information, presence of metabolic comorbidities, antiviral therapy status, laboratory parameters—alanine aminotransferase (ALT), alkaline phosphatase (ALP), total bilirubin (TB), albumin, platelet count, hepatitis B e antigen (HBeAg), and HBV DNA viral load—as well as imaging findings related to hepatic steatosis and cirrhosis. Liver cirrhosis was defined based on imaging features suggestive of advanced fibrosis, including a small, nodular liver with or without splenomegaly or ascites on ultrasonography, CT, or MRI, or a liver stiffness measurement >17 kPa as determined by transient elastography [30].

### Study outcome

The primary outcome of this study was to assess the impact of CMRFs on the risk of HCC in patients with CHB. The incidence of HCC, identified through the Songklanagarind Hospital Cancer Registry. HCC diagnosis followed established criteria from the American Association for the Study of Liver Diseases (AASLD) [31] or the European Association for the Study of the Liver (EASL) [32], requiring the presence of a lesion ≥1 cm with characteristic radiologic features, arterial phase hyperenhancement with washout in the venous or delayed phases, on either CT or MRI. The censor date for HCC diagnosis was set as December 31, 2024. Secondary outcomes included evaluating the risk of HCC across predefined subgroups, such as patients with cirrhosis or those receiving antiviral therapy. Furthermore, the study aimed to compare risk prediction performance using the aMAP score [18, 33], which has been proposed as a stratification tool for HCC risk within this patient population.

### Statistical analyses

Quantitative variables were summarized as mean (standard deviation [SD]) or median (interquartile range [IQR]), depending on data distribution. Categorical variables were reported as frequencies and percentages. Time-to-event analyses were conducted using the Kaplan-Meier method, with survival differences between groups assessed by the log-rank test. Cox proportional hazards regression models were employed to identify predictors of HCC, with adjusted hazard ratios (aHR) and 95% confidence intervals (CI) reported. A two-sided p-value < 0.05 was considered statistically significant. As a sensitivity analysis, we excluded patients with baseline cirrhosis at index to better isolate the effect of MASLD and the same analysis was performed. Statistical analyses were performed using R version 4.2.3.

## Results

### Baseline characteristics of the study cohort

A total of 4,944 patients with CHB were included in the analysis. The median age at baseline was 50.6 years (IQR, 38.8–60.7), and 55.9% were male. Based on BMI classification, 14.5% of patients were overweight (BMI 23.0–24.9 kg/m²), and 27.2% were classified as obese (BMI ≥ 25.0 kg/m²). Regarding CMRFs profiles, 18.1% had HT, 14.0% had DM, and 25.5% had DLP. Hepatic steatosis was identified in 20.6% of patients, and 14.4% met diagnostic criteria for MASLD. Cirrhosis was diagnosed in 20.1% of the cohort. Baseline clinical characteristics stratified by HCC status are summarized in Table 1.

**Table 1:** Baseline demographics and patient characteristics of the study cohort.

Variable	Overall (n = 4,944^*1*^)	No HCC (n = 4,793^*1*^)	HCC (n = 151^*1*^)	p-value
Age (years)	50.59 (38.83-60.74)	50.23 (38.47-60.48)	58.43 (51.72-65.55)	<0.001^*2*^
Male, n (%)	2,762 (55.9)	2,663 (55.6)	99 (65.6)	0.015^*3*^
Weight (kg)	61.10 (53.20-70.00)	61.00 (53.00-70.00)	64.80 (56.00-73.44)	0.027^*2*^
Height (cm)	162.00 (155.50-168.00)	162.00 (156.00-168.00)	163.00 (155.00-169.50)	0.6^*2*^
BMI (kg/m^2^)	23.28 (20.72-26.40)	23.21 (20.69-26.34)	24.75 (21.87-27.29)	0.005^*2*^
Antiviral therapy, n (%)	1,172 (23.7)	1,089 (22.7)	83 (55.0)	<0.001^*3*^
Hypertension, n (%)	893 (18.1)	828 (17.3)	65 (43.0)	<0.001^*3*^
Dyslipidemia, n (%)	1,259 (25.5)	1,218 (25.4)	41 (27.1)	0.5^*3*^
with statin	463 (9.4)	451 (9.4)	12 (7.9)	
without statin	796 (16.1)	767 (16.0)	29 (19.2)	
Diabetes mellitus, n (%)	693 (14.0)	658 (13.7)	35 (23.2)	0.004^*3*^
Prediabetes, n (%)	438 (8.9)	425 (8.9)	13 (8.6)	
Obese, n (%)	1,347 (27.2)	1,277 (26.6)	70 (46.4)	<0.001^*3*^
Overweight, n (%)	719 (14.5)	693 (14.5)	26 (17.2)	
No. of CMRFs				<0.001^*4*^
none	2,758 (55.8)	2,715 (56.6)	43 (28.5)	
1	1,553 (31.4)	1,494 (31.2)	59 (39.1)	
2	519 (10.5)	483 (10.1)	36 (23.8)	
3	114 (2.3)	101 (2.1)	13 (8.6)	
MASLD, n (%)	714 (14.4)	702 (14.6)	12 (7.9)	0.021^*3*^
Hepatic steatosis, n (%)	1,017 (20.6)	1,000 (20.9)	17 (11.3)	0.004^*3*^
CAP, dB/m	210.50 (180.00-246.00)	210.00 (180.00-247.00)	219.50 (202.00-234.00)	0.5^2^
Method CAP, n (%) Imaging, n (%)	37 (0.7) 980 (19.8)	37 (0.8) 963 (20.1)	0 (0.0) 17 (11.3)	0.013^5^
Cirrhosis, n (%)	992 (20.1)	867 (18.1)	125 (82.8)	<0.001^*3*^
ALT, *U/L*	29.00 (17.00-54.00)	28.00 (17.00-53.00)	45.00 (30.00-70.00)	0.8^*2*^
ALP, *U/L*	79.00 (61.00-109.00)	78.00 (61.00-108.00)	101.00 (73.00-135.00)	0.6^*2*^
Total bilirubin, *mg/dL*	0.54 (0.36-0.88)	0.54 (0.36-0.86)	0.80 (0.46-1.32)	0.5^*2*^
Albumin, *g/dL*	3.97 (0.72)	3.98 (0.72)	3.66 (0.69)	<0.001^*2*^
Platelet, x10^6^*/mm*^*3*^	239.65 (117.57)	242.80 (117.50)	145.80 (72.71)	<0.001^*2*^
HBeAg positive, n (%)	580 (11.7)	552 (11.5)	28 (18.5)	0.008^*3*^
HBV DNA, *log IU/mL*	3.16 (1.91-5.54)	3.13 (1.89-5.44)	5.48 (3.02-6.44)	<0.001^*2*^
Death, n (%)	1,488 (30.1)	1,396 (29.1)	92 (60.9)	<0.001^*3*^
Year of diagnosis				<0.001^*3*^
2011	490 (9.9)	460 (9.6)	30 (19.9)	
2012	466 (9.4)	449 (9.4)	17 (11.3)	
2013	480 (9.7)	456 (9.5)	24 (15.9)	
2014	447 (9.0)	434 (9.1)	13 (8.6)	
2015	391 (7.9)	377 (7.9)	14 (9.3)	
2016	477 (9.6)	462 (9.6)	15 (9.9)	
2017	415 (8.4)	407 (8.5)	8 (5.3)	
2018	450 (9.1)	437 (9.1)	13 (8.6)	
2019	473 (9.6)	467 (9.7)	6 (4.0)	
2020	460 (9.3)	455 (9.5)	5 (3.3)	
2021	395 (8.0)	389 (8.1)	6 (4.0)	

^1^n (%).

^2^Two Sample t-test.

^3^Pearson’s Chi-squared test.

^4^Fisher’s Exact test for Count Data with simulated p-value (based on 100000 replicates).

^5^Fisher’s Exact test.

Abbreviations: CHB, chronic hepatitis B; BMI, body mass index; HT, hypertension; DLP, dyslipidemia; DM, diabetes mellitus; MASLD, metabolic dysfunction-associated steatotic liver disease; CAP, controlled attenuation parameter; ALT, alanine aminotransferase; ALP, alkaline phosphatase; TB, total bilirubin; HBeAg, hepatitis B e antigen; HBV, hepatitis B virus.

Compared with patients without HCC, those who developed HCC were older (median age, 58.4 vs. 50.2 years; p < 0.001), more frequently male (65.6% vs. 55.6%; p = 0.015), and had a higher BMI (24.8 vs. 23.2 kg/m²; p = 0.005). A significantly greater proportion of patients with HCC received antiviral therapy (55.0% vs. 22.7%; p < 0.001) and had comorbid conditions, including HT (43.0% vs. 17.3%; p < 0.001), DM (23.2% vs. 13.7%; p = 0.004), and obesity (46.4% vs. 26.6%; p < 0.001). Cirrhosis prevalence was markedly higher in the HCC group compared to those without HCC (82.8% vs. 18.1%; p < 0.001).

Although DLP was common in both groups (27.1% in HCC vs. 25.4% in non-HCC, p = 0.53), a higher proportion of HCC patients did not receive statin therapy (19.2%). Notably, hepatic steatosis and MASLD were less prevalent among patients with HCC compared to those without (11.3% vs. 20.9%, p = 0.004 and 7.9% vs. 14.6%, p = 0.021, respectively). The distribution of CMRFs differed significantly by HCC status (p < 0.001), with a greater proportion of patients with HCC having ≥ 2 CMRFs.

### Cumulative incidence of HCC in the entire cohort

**Fig 1:**
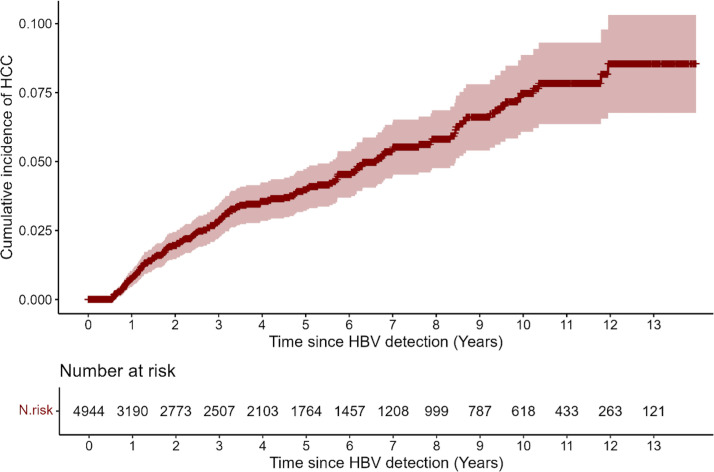
Nelson-Aalen plot depicting the cumulative incidence of hepatocellular carcinoma (HCC) over time in the overall cohort of patients with chronic hepatitis B (CHB).

During follow-up, 151 patients (3.1%) developed HCC. The cumulative incidence of HCC increased progressively over time, as illustrated by the Nelson-Aalen curve (Fig 1). At 5 and 10 years, the cumulative incidence was 4.0% (95% CI: 3.2–4.7) and 7.5% (95% CI: 6.1–8.9), respectively.

### Cumulative incidence of HCC stratified by CMRFs and hepatic steatosis

**Fig 2:**
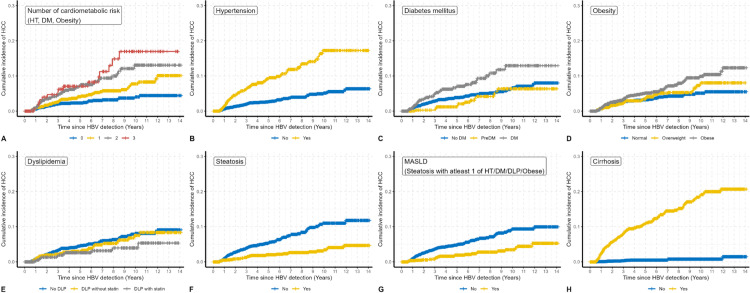
Nelson-Aalen plots showing cumulative HCC incidence stratified by individual risk factors in the entire cohort, including the number of cardiometabolic risk factors (CMRFs), hypertension (HT), diabetes mellitus (DM), obesity, dyslipidemia (with and without statin use), hepatic steatosis, metabolic dysfunction-associated steatotic liver disease (MASLD), and cirrhosis.

Subgroup analyses revealed that cirrhosis was the strongest predictor of HCC incidence, with a markedly higher 10-year cumulative incidence compared to non-cirrhotic patients (Fig 2H). Among individual CMRFs, obesity was associated with an increased HCC incidence, reaching approximately 10.3% (95% CI: 7.6–13.1) at 10 years. Patients with DM and HT exhibited intermediate increases in cumulative incidence, approximately 13.0% (95% CI: 7.9–18.0) and 17.4% (95% CI: 12.3–22.4), respectively, at 10 years (Figs 2B–D). DLP without statin therapy was also linked to a higher 10-year cumulative HCC incidence of 7.6% (95% CI: 4.4–10.8) (Fig 2E), whereas patients with DLP receiving statins demonstrated a significantly lower incidence of 4.0% (95% CI: 1.4–6.6).

Notably, hepatic steatosis and MASLD were not associated with an increased risk of HCC. The 10-year cumulative incidence of HCC in patients with MASLD was approximately 2.7% (95% CI: 1.0–4.4), compared to 8.8% (95% CI: 7.1–10.6) in those without MASLD (Fig 2G). Similarly, patients with hepatic steatosis had a 10-year cumulative incidence of 2.6% (95% CI: 1.2–4.0) versus 9.5% (95% CI: 7.6–11.4) in those without steatosis (Fig 2F). Moreover, patients with three or more CMRFs exhibited the highest 10-year cumulative incidence, reaching 17.7% (95% CI: 8.1–27.3), compared with 4.0% (95% CI: 2.5–5.4) in those without any CMRFs (Fig 2A).

### HCC incidence stratified by MASLD status and aMAP score

**Fig 3:**
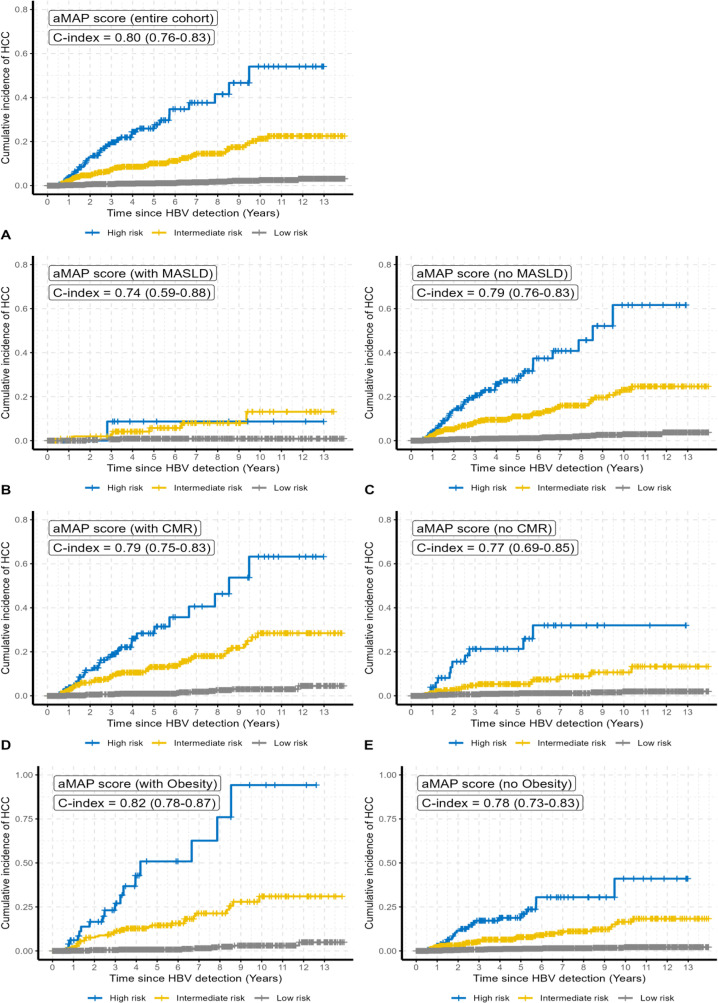
Nelson-Aalen plots illustrating cumulative HCC incidence stratified by aMAP risk score categories (low, intermediate, high), with subgroup analyses based on MASLD status, presence of CMRFs, and obesity. aMAP, Age-Male-ALBI-Platelet score; MASLD, metabolic ysfunction-associated steatotic liver disease; CMR, cardiometabolic risk.

Stratification by MASLD status demonstrated that patients with MASLD exhibited a lower 10-year cumulative incidence of HCC across all aMAP score risk categories compared to those without MASLD (Fig 3, Table 2). Specifically, among patients with MASLD, the 10-year cumulative incidence of HCC was 0.9% (95% CI: 0.0–1.9) in the low-risk group, 12.9% (95% CI: 1.1–24.8) in the intermediate-risk group, and 8.3% (95% CI: 0.0–24.7) in the high-risk group. Conversely, patients without MASLD had substantially higher 10-year HCC incidences of 3.0% (95% CI: 1.5–4.4), 23.1% (95% CI: 16.0–30.1), and 60.6% (95% CI: 32.5–88.7) in the low-, intermediate-, and high-risk groups, respectively.

**Table 2:** Cumulative Incidence of HCC, Stratified by Cohort and aMAP Score Risk Group.

Cohort								
aMAP score	Time	Entire cohort	With MASLD	No MASLD	With CMRFs	No CMRFs	With obesity	No obesity
low	5 yr	1.0 (0.5-1.5)	0.6 (0.0-1.4)	1.2 (0.5-1.8)	1.0 (0.3-1.7)	1.0 (0.3-1.7)	0.8 (0.0- 1.5)	1.1 (0.5-1.8)
intermediate	5 yr	10.0 (7.2-12.9)	6.1 (1.8-10.4)	11.5 (8.0-15.1)	13.0 (8.8-17.2)	5.3 (2.1-8.5)	14.5 (8.6-20.4)	7.8 (4.7-10.9)
high	5 yr	25.7 (16.6-34.8)	15.0 (0.0-35.9)	26.6 (16.8-36.4)	28.0 (16.2-39.8)	21.1 (7.2-35.0)	49.8 (21.1-78.4)	18.7 (10.1-27.2)
low	10 yr	2.5 (1.3-3.6)	0.6 (0.0-1.4)	3.5 (1.8-5.3)	3.0 (1.2-4.8)	2.0 (0.6-3.4)	3.0 (0.9- 5.2)	2.2 (0.9-3.4)
intermediate	10 yr	21.2 (15.0-27.3)	16.3 (6.8-25.8)	23.2 (15.3-31.1)	28.2 (18.7-37.7)	10.6 (4.4-16.8)	30.8 (18.1-43.4)	16.4 (9.7-23.2)
high	10 yr	52.8 (29.9-75.8)	15.0 (0.0-35.9)	61.6 (31.8-91.4)	61.2 (30.6-91.7)	31.2 (11.4-50.9)	90.0 (35.6-144.5)	40.2 (15.9-64.5)

Abbreviations: aMAP, Age-Male-ALBI-Platelet score; MASLD, metabolic dysfunction-associated steatotic liver disease; CMRFs, cardiometabolic risk factors; CHB, chronic hepatitis B.

This pattern persisted at the 5-year time point, where patients with MASLD had HCC incidences of 0.9% (95% CI: 0.0–1.9), 5.7% (95% CI: 0.6–10.8), and 8.3% (95% CI: 0.0–24.7) across the low-, intermediate-, and high-risk categories, respectively. In contrast, the corresponding 5-year incidences in non-MASLD patients were 1.0% (95% CI: 0.4–1.6), 11.0% (95% CI: 7.7–14.3), and 27.3% (95% CI: 17.4–37.2).

### HCC incidence stratified by CMRFs and aMAP score

Stratification by the presence of CMRFs revealed similar trends. Among patients with CMR, the 10-year cumulative incidence of HCC was 3.0% (95% CI: 1.2–4.8) in the low-risk aMAP group, 28.3% (95% CI: 18.8–37.9) in the intermediate-risk group, and 61.9% (95% CI: 30.9–93.0) in the high-risk group. In contrast, patients without CMRFs had corresponding 10-year incidences of 2.0% (95% CI: 0.6–3.4), 10.6% (95% CI: 4.4–16.8), and 31.5% (95% CI: 11.3–51.6), respectively (Fig 3, Table 2). At 5 years, patients with CMRFs demonstrated HCC incidence rates of 1.0% (95% CI: 0.3–1.7), 13.0% (95% CI: 8.8–17.2), and 28.2% (95% CI: 16.2–40.2) in the low-, intermediate-, and high-risk groups, respectively. Among patients without CMRFs, the 5-year incidences were 1.0% (95% CI: 0.3–1.7), 5.3% (95% CI: 2.1–8.5), and 21.1% (95% CI: 7.2–35.0), respectively.

### HCC incidence stratified by obesity status and aMAP score

When stratified by obesity status, the 10-year cumulative incidence of HCC increased across all aMAP risk categories. Among patients with obesity, the 10-year cumulative HCC incidence was 3.0% (95% CI: 0.9–5.2) in the low-risk group, 30.8% (95% CI: 18.1–43.4) in the intermediate-risk group, and 90.0% (95% CI: 35.6–144.5) in the high-risk group. In contrast, non-obese patients exhibited corresponding 10-year incidences of 2.2% (95% CI: 0.9–3.4), 16.4% (95% CI: 9.7–23.2), and 40.2% (95% CI: 15.9–64.5), respectively (Fig 3, Table 2). At 5 years, cumulative HCC incidences among obese patients were 0.8% (95% CI: 0.0–1.5), 14.5% (95% CI: 8.6–20.4), and 49.8% (95% CI: 21.1–78.4) in the low-, intermediate-, and high-risk groups, respectively. Non-obese patients had corresponding incidences of 1.1% (95% CI: 0.5–1.8), 7.8% (95% CI: 4.7–10.9), and 18.7% (95% CI: 10.1–27.2).

### Performance of the aMAP score for HCC risk stratification in metabolically burdened CHB patients

The aMAP score demonstrated robust performance in stratifying HCC risk across the overall cohort as well as within the CMRFs, MASLD, and obesity subgroups. The model achieved a concordance index (C-index) of 0.74 in patients with MASLD, 0.79 in those with CMRFs, and 0.82 in patients with obesity, effectively discriminating low-, intermediate-, and high-risk groups (Fig 3). These results support the applicability of the aMAP score for HCC risk stratification in CHB populations with metabolic comorbidities.

### Independent risk factors for HCC development identified by multivariable cox regression analysis

**Fig 4:**
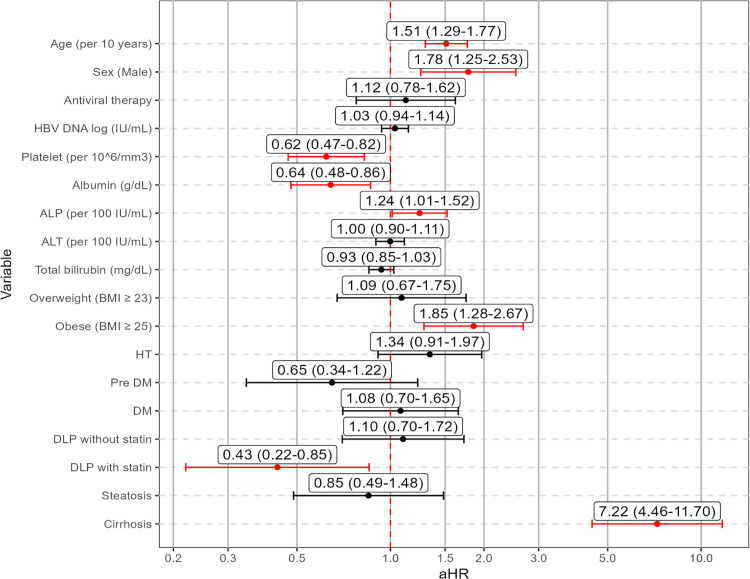
Forest plot summarizing multivariable Cox regression analysis identifying factors independently associated with HCC development. HCC, hepatocellular carcinoma; aHR, adjusted hazard ratio; ALP, alkaline phosphatase; ALT, alanine aminotransferase; DLP, dyslipidemia; HT, hypertension; DM, diabetes mellitus; CHB, chronic hepatitis **B.**

Multivariable Cox regression analysis identified several independent risk factors for HCC development, as detailed in Table 3 and summarized in Fig 4. Increasing age (per 10-year increment; aHR 1.51, 95% CI: 1.29–1.77; p < 0.001), male sex (aHR 1.78, 95% CI: 1.25–2.53; p = 0.006), elevated ALP (aHR 1.24, 95% CI: 1.01–1.52; p = 0.042), obesity (aHR 1.85, 95% CI: 1.28–2.67; p = 0.001), and cirrhosis (aHR 7.22, 95% CI: 4.46–11.70; p < 0.001) were independently associated with increased HCC risk. Conversely, higher platelet count (aHR 0.62, 95% CI: 0.47–0.82; p = 0.001), higher serum albumin (aHR 0.64, 95% CI: 0.48–0.86; p = 0.003), and DLP with statin use (aHR 0.43, 95% CI: 0.22–0.85; p = 0.015) were significantly associated with reduced HCC risk. After adjustment, antiviral therapy, HBV DNA level, ALT, TB, hypertension, and hepatic steatosis were not significantly associated with HCC risk.

**Table 3:** Cox Regression Analysis for Factors Associated with HCC Development.

Variable	Univariable		Multivariable	
	HR (95% CI)	*P-value*	HR (95% CI)	*P-value*
Demographic				
Age (per 10 years)	1.64 (1.46-1.85)	<0.001	1.51 (1.29-1.77)	<0.001
Gender, male vs female	1.77 (1.26-2.47)	<0.010	1.78 (1.25-2.53)	<0.010
Antiviral therapy, yes vs no	3.51 (2.55-4.84)	<0.001	1.12 (0.78-1.62)	0.545
Laboratory				
HBV DNA (*log IU/mL*)	1.16 (1.08-1.25)	<0.001	1.03 (0.94-1.14)	0.518
Platelet (x10^6^/*mm*^*3*^)	0.27 (0.21-0.34)	<0.001	0.62 (0.47-0.82)	<0.010
Albumin (*g/dL*)	0.39 (0.32-0.47)	<0.001	0.64 (0.48-0.86)	<0.010
ALP (*U/L*)	1.24 (1.10-1.40)	<0.010	1.24 (1.01-1.52)	<0.050
ALT (*U/L*)	0.99 (0.92-1.07)	0.824	1.00 (0.90-1.11)	0.962
Total bilirubin (*mg/dL*)	1.05 (1.00-1.10)	0.072	0.93 (0.85-1.03)	0.156
Cardiometabolic risk factors				
Overweight (BMI ≥ 23)	1.32 (0.83-2.11)	0.244	1.09 (0.67-1.75)	0.735
Obese (BMI ≥ 25)	1.84 (1.29-2.62)	<0.001	1.85 (1.28-2.67)	<0.010
Hypertension	3.11 (2.25-4.29)	<0.001	1.34 (0.91-1.97)	0.139
Prediabetes	0.72 (0.40-1.28)	0.274	0.65 (0.34-1.22)	0.183
Diabetes mellitus	1.83 (1.24-2.68)	<0.010	1.08 (0.70-1.65)	0.734
Dyslipidemia without statin	0.86 (0.57-1.29)	0.479	1.10 (0.70-1.72)	0.685
Dyslipidemia with statin	0.58 (0.32-1.05)	0.080	0.43 (0.22-0.85)	<0.050
Liver-related				
Hepatic steatosis, yes vs no	0.31 (0.19-0.51)	<0.001	0.85 (0.49-1.48)	0.567
Cirrhosis, yes vs no	16.99 (11.14-25.93)	<0.001	7.22 (4.46-11.70)	<0.001

Abbreviations: HR, hazard ratio; CI, confidence interval; aHR, adjusted hazard ratio; ALP, alkaline phosphatase; ALT, alanine aminotransferase; TB, total bilirubin; DLP, dyslipidemia; DM, diabetes mellitus; HT, hypertension.

### Sensitivity analysis excluding baseline cirrhosis

After excluding baseline cirrhosis, 26 patients developed HCC during follow-up. In multivariable Cox regression, male sex remained significantly associated with HCC (aHR 3.48, 95% CI 1.14–10.59; p < 0.05), whereas age (per 10 years; aHR 1.40, 95% CI 0.95–2.07; p = 0.080) and diabetes mellitus (aHR 2.98, 95% CI 0.95–9.41; p = 0.059) showed borderline associations (S1 Fig). The hepatic steatosis was directionally consistent but not statistically significant, consistent with reduced event counts. No HCC events occurred in the aMAP high-risk group in this subset. Overall discrimination was modestly lower than in the primary analysis (C-index ≈ 0.70), as shown in S2 Fig.

## Discussion

In this large retrospective cohort study of 4,944 patients with CHB conducted at a tertiary center in southern Thailand, we found that the presence of CMRFs was significantly associated with an increased risk of HCC. Cirrhosis was identified as the strongest predictor of HCC, followed by obesity and male sex. Conversely, DLP with statin use, higher platelet count, and higher serum albumin levels were independently associated with a reduced risk of HCC development. Furthermore, patients with a greater number of CMRFs exhibited a progressively higher cumulative incidence of HCC over time. The 10-year cumulative incidence of HCC in our cohort was 7.5%, consistent with prior studies of CHB patients with metabolic dysfunction [34]. Additionally, the aMAP score demonstrated effective risk stratification for HCC across subgroups defined by obesity, CMRFs, MASLD, and including in the non-cirrhotic sensitivity cohort, with robust discrimination observed in these metabolically burdened populations.

Our study identified that HCC was more prevalent among older males with obesity, consistent with prior research demonstrating the influence of age, sex, and obesity on HCC risk [7,35–37]. Among the risk factors analyzed, cirrhosis was the most significant, conferring an approximately sevenfold increased risk compared to non-cirrhotic patients. Additionally, DLP with statin use was associated with a reduced risk of HCC, whereas DLP without statin therapy showed no significant effect. This finding aligns with recent evidence supporting a chemopreventive role for statins in reducing HCC risk [38,39].

The presence of CMRFs, including DM, HT, and obesity, was independently associated with increased HCC risk [10]. Moreover, the risk appeared cumulative, as patients with multiple CMRFs exhibited a higher incidence of HCC, consistent with previous studies [7,16,40–43]. In contrast, isolated hepatic steatosis did not significantly increase HCC risk in our cohort, corroborating reports of no elevated HCC risk with hepatic steatosis during long-term follow-up in CHB [14,44–46]. This may reflect dynamic changes in liver fat content over time, as fibrosis progression is often accompanied by reduced hepatic fat, resulting in an inverse association between steatosis and mortality [47]. Furthermore, the “burnout” phenomenon observed in advanced cirrhosis may account for the loss of hepatic fat, indicating advanced fibrosis rather than a protective effect. Lower CAP values, reflecting diminished liver fat, have been linked to increased fibrosis and HCC risk [48].

Interestingly, antiviral therapy and HBV viral load levels did not demonstrate a direct correlation with HCC risk in our cohort. One potential explanation is that early initiation of antiviral therapy may confer greater protection, whereas initiation at advanced cirrhotic stages may not effectively reduce HCC risk. Additionally, lower HBV DNA levels were independently associated with HBsAg seroclearance, consistent with prior findings indicating that HBsAg loss, either spontaneously or during antiviral therapy, serves as a surrogate marker of functional cure in CHB [49]. The significant association between MASLD and a higher rate of HBsAg seroclearance further supports this notion, suggesting that metabolic components may facilitate HBsAg loss and contribute to improved prognosis [49].

Our findings also align with emerging evidence indicating that MASLD is not independently associated with increased HCC risk after adjustment for cirrhosis [50]. This concurs with recent data demonstrating that MAFLD is linked to increased HCC risk primarily in the absence of advanced liver disease [50].

Moreover, our analysis demonstrated that the aMAP score effectively stratifies patients into low-, intermediate-, and high-risk groups for HCC development. Consistent with previous validation studies [18,33], patients with a low aMAP score exhibited low 5- and 10-year cumulative incidences of HCC. In contrast, those with intermediate to high aMAP scores showed significantly elevated HCC incidence, highlighting the utility of the aMAP score in identifying high-risk individuals who may benefit from enhanced HCC surveillance. Importantly, no HCC events occurred in the high-risk aMAP category within the non-cirrhotic subset, likely reflecting limited statistical power. Nevertheless, the aMAP score maintained modest discrimination (C-index ≈ 0.70) in the sensitivity analysis, supporting its utility for HCC risk stratification even in the absence of cirrhosis. Taken together, these findings suggest that the relationship between metabolic factors, hepatic steatosis, and HCC risk in CHB is complex and context-dependent. Incorporating metabolic risk assessment into existing surveillance models may improve HCC risk prediction and inform clinical decision-making, particularly in HBV-endemic regions experiencing a rising prevalence of metabolic syndrome.

Despite the strengths of our study, including a large cohort size and comprehensive data analysis, several limitations should be acknowledged. First, the retrospective design inherently limits the ability to infer causality and is susceptible to potential residual confounding from unmeasured variables. Important lifestyle factors, such as the quantity and pattern of alcohol consumption, physical activity, and dietary habits, were not captured, which may influence HCC risk and metabolic status. Second, hepatic steatosis assessment relied primarily on non-invasive measures without histological confirmation, which remains the gold standard for accurately characterizing liver fat content and differentiating steatosis severity. Moreover, advanced imaging techniques such as magnetic resonance imaging-proton density fat fraction (MRI-PDFF), which provide precise quantitative evaluation of liver fat, were not available in this cohort, potentially limiting the accuracy of fat quantification and its correlation with HCC risk. Third, despite adjustment for all available clinical and treatment-related variables, residual confounding due to antiviral therapy adherence cannot be fully excluded. Adherence patterns, and treatment interruptions, together with virological response, all factors previously recognized to impact HCC risk. Fourth, confounding by indication may affect the observed association between antiviral therapy and HCC, as patients with more advanced disease were more likely to receive treatment. Additionally, it is noteworthy that only 3.1% of patients in the cohort developed HCC during the follow-up period. While this incidence is consistent with prior reports in chronic hepatitis B populations, it underscores the relatively low absolute risk of HCC and highlights the importance of robust risk stratification tools to identify high-risk individuals who would benefit most from surveillance. Given these limitations, prospective longitudinal studies are warranted to validate the associations observed and to elucidate the underlying biological mechanisms linking cardiometabolic risk factors and HCC development. Furthermore, randomized controlled trials investigating the efficacy of statin therapy in chemoprevention could provide critical insights into its potential role in reducing HCC incidence in at-risk populations.

## Conclusions

In summary, cirrhosis, male sex, and obesity are key risk factors for HCC in patients with CHB, while dyslipidemia treated with statins, higher platelet counts, and serum albumin levels are associated with reduced risk. The risk of HCC increases with the number of cardiometabolic risk factors present. The aMAP score effectively stratifies HCC risk in patients with obesity, CMRFs, and MASLD, aiding in identifying those who may benefit from closer monitoring. Incorporating cardiometabolic assessments into HCC risk evaluation could improve surveillance strategies. Additionally, the protective effect of statins suggests that managing metabolic factors may help lower HCC risk in this population.

## Supporting information

S1 Fig Forest plot summarizing multivariable Cox regression analyses identifying factors independently associated with HCC development in a sensitivity analysis excluding patients with baseline cirrhosis. HCC, hepatocellular carcinoma; aHR, adjusted hazard ratio; ALP, alkaline phosphatase; ALT, alanine aminotransferase; DLP, dyslipidemia; HT, hypertension; DM, diabetes mellitus; CHB, chronic hepatitis B.
(TIFF)

S2 Fig Nelson-Aalen plots illustrating cumulative HCC incidence stratified by aMAP risk score categories (low, intermediate, high), with subgroup analyses based on MASLD status, presence of CMRFs, and obesity, derived from a sensitivity analysis excluding baseline cirrhosis. aMAP, Age-Male-ALBI-Platelet score; MASLD, metabolic dysfunction-associated steatotic liver disease; CMRFs, cardiometabolic risk factors.
(TIFF)

## Abbreviations

aHR: Adjusted Hazard Ratio
ALBI: Albumin-Bilirubin
ALP: Alkaline Phosphatase
ALT: Alanine Aminotransferase
aMAP: Age, Male, ALBI, Platelet Score,
BMI: : Body Mass Index,
CAP: : Controlled Attenuation Parameter,
CHB: : Chronic Hepatitis B,
CI: : Confidence Interval,
CMRFs: : Cardiometabolic Risk Factors,
CT: : Computed Tomography,
DBP: : Diastolic Blood Pressure,
DLP: : Dyslipidemia,
DM: : Diabetes Mellitus,
HbA1c: : Hemoglobin A1c,
HBeAg: : Hepatitis B e Antigen,
HBV: : Hepatitis B Virus,
HBV DNA: : Hepatitis B Virus Deoxyribonucleic Acid,
HCC: : Hepatocellular Carcinoma,
HDL: : High-Density Lipoprotein,
HT: : Hypertension,
IQR: : Interquartile Range,
LDL: : Low-Density Lipoprotein,
MASLD: : Metabolic Dysfunction-Associated Steatotic Liver Disease,
MRI: : Magnetic Resonance Imaging,
NAFLD: : Non-Alcoholic Fatty Liver Disease,
PAGE-B: : Platelet Age Gender-HBV,
SBP: : Systolic Blood Pressure,
SD: : Standard Deviation,
TB: : Total Bilirubin,
T2DM: : Type 2 Diabetes Mellitus.
