# Supplementary figures and images for: Impact of cardiometabolic risk factors on hepatocellular carcinoma incidence in patients with chronic hepatitis B: A retrospective cohort study
Source: PLoS One. 2026 Jan 23;21(1):e0341366. doi: 10.1371/journal.pone.0341366 (PMC12829800; doi:10.1371/journal.pone.0341366)

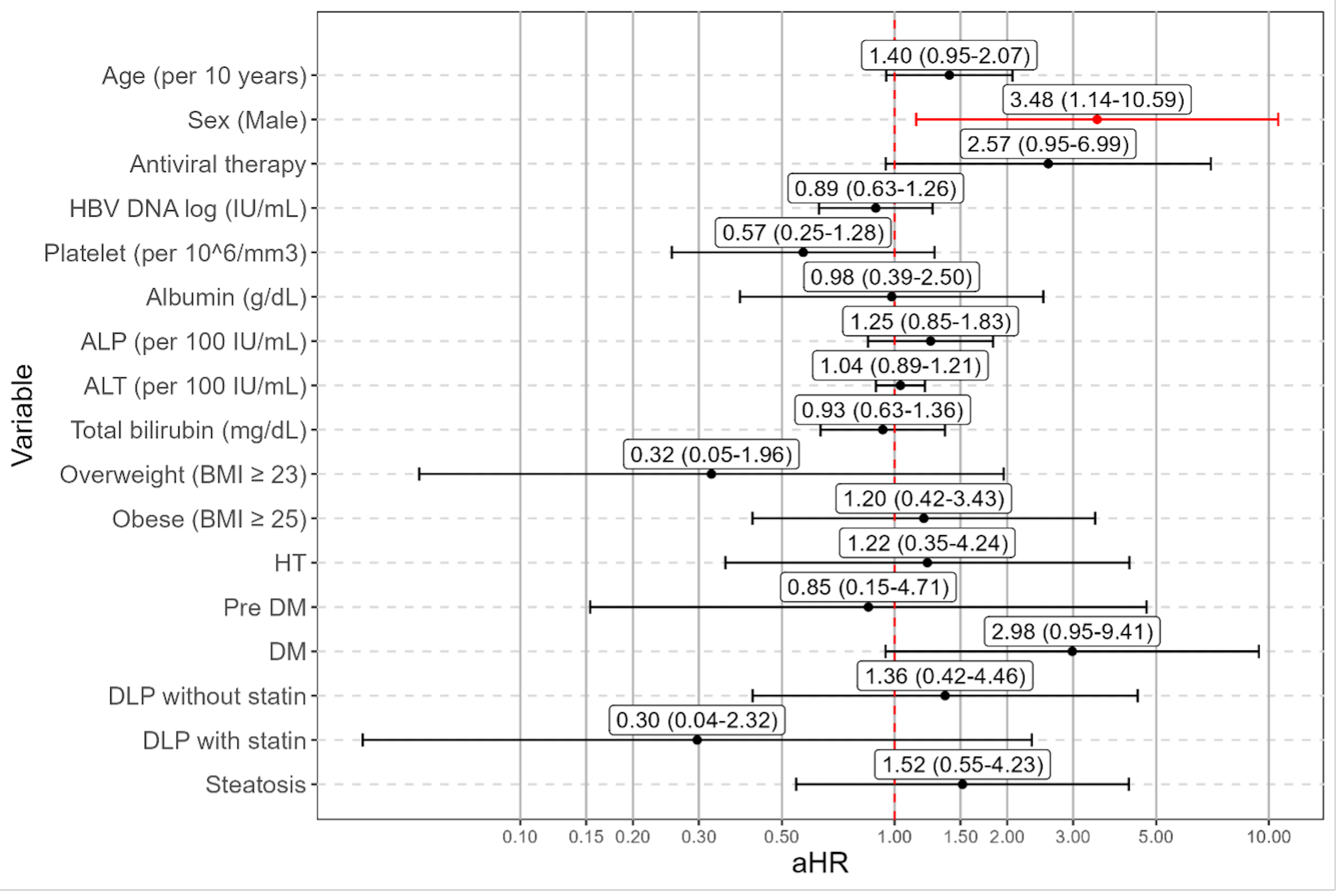

Supplement: S1 Fig — HCC, hepatocellular carcinoma; aHR, adjusted hazard ratio; ALP, alkaline phosphatase; ALT, alanine aminotransferase; DLP, dyslipidemia; HT, hypertension; DM, diabetes mellitus; CHB, chronic hepatitis B. (TIFF) [file pone.0341366.s001.tiff]

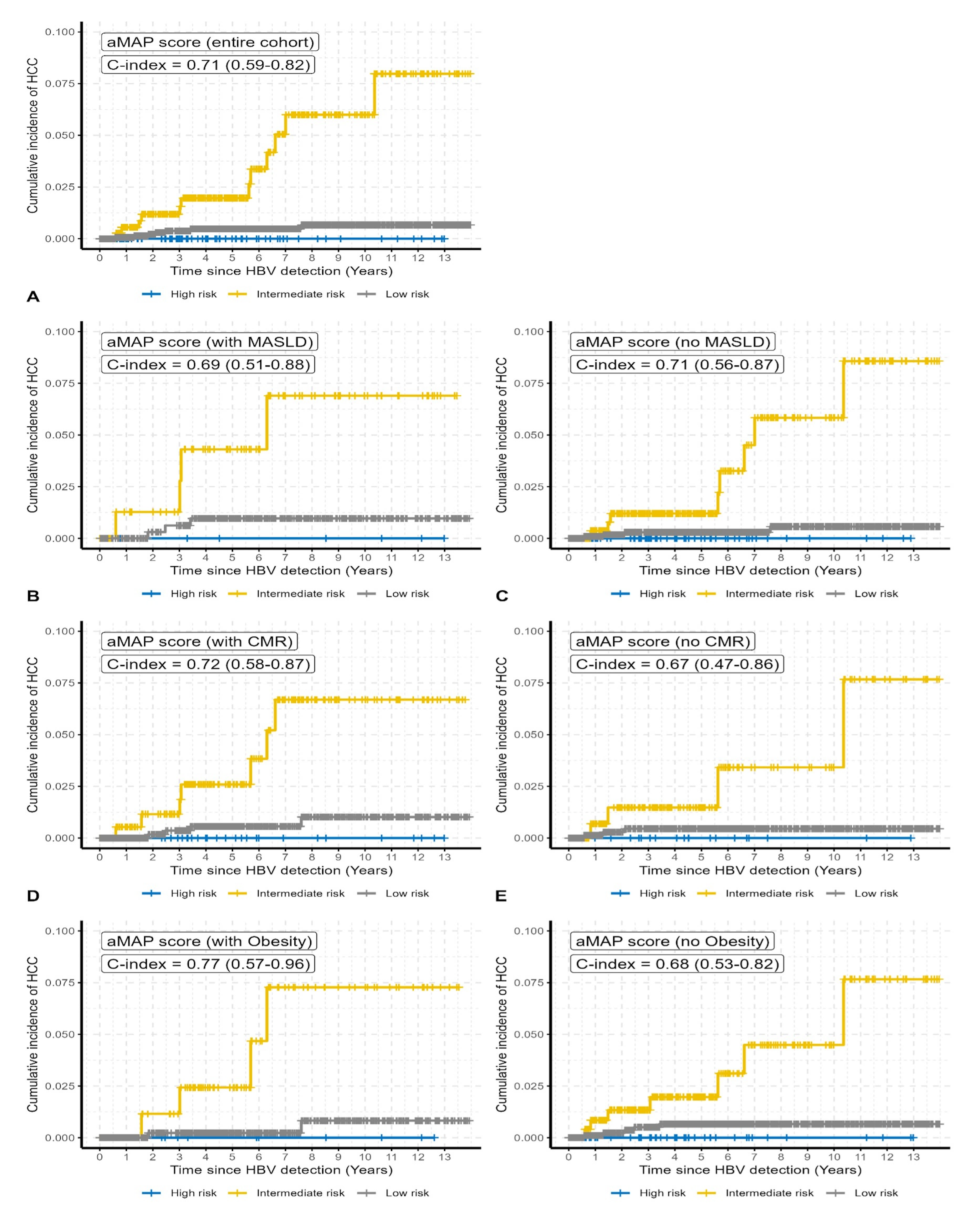

Supplement: S2 Fig — aMAP, Age-Male-ALBI-Platelet score; MASLD, metabolic dysfunction-associated steatotic liver disease; CMRFs, cardiometabolic risk factors. (TIFF) [file pone.0341366.s002.tiff]
